# Relationships among leaf, stem, and root traits of *Pseudotsuga sinensis* under karst rocky desertification habitats, southwest China

**DOI:** 10.3389/fpls.2026.1792414

**Published:** 2026-03-19

**Authors:** Wangjun Li, Fangwen He, Lan Li, Shun Zou, Bin He, Xiaolong Bai, Yurong Yang

**Affiliations:** 1 Guizhou University of Engineering Science, Bijie, China; 2 State Environmental Protection Key Laboratory of Wetland Ecology and Vegetation Restoration, School of Environment, Northeast Normal University, Changchun, China

**Keywords:** ecological strategies, plant functional traits, *Pseudotsuga sinensis*, rocky desertification, trade-off

## Abstract

**Introduction:**

Plants adapt to different environmental conditions through resource allocation and optimization across different organs (leaves, stems, and roots). Across different ecosystems or communities, these organs may coordinate, trade off, or operate independently to adapt to habitat environmental conditions. However, in rocky desertification habitats, the specific inter-organ relationships during resource allocation and optimization remain unclear due to limited research.

**Methods:**

This study examined 30 trait characteristics of leaves, stems, and fine roots of Pseudotsuga sinensis, a tree species adapted to rocky desertification environments, to investigate the associations among organs.

**Results:**

Principal component analysis (PCA) revealed that leaf traits were structured along orthogonal axes representing defense (PCA 1) and nutrient strategy (PCA 2). Stem traits were organized along axes related to water acquisition and transport (PCA 1) and nutrient strategy (PCA 2), while fine root traits aligned with defense (PCA 1) and nutrient strategy (PCA 2). Correlation analysis indicated a significant negative relationship between leaves and fine roots in Pseudotsuga sinensis, suggesting a trade-off in defense investment between these organs in response to rocky desertification conditions. Non-significant correlations between stems and roots, as well as between leaves and stems, imply independent defense and resource strategies in these organs.

**Conclusions:**

The findings revealed that the linkage mechanisms and strategies between leaves and roots in plants adapting to rocky desertification habitats, provide insights for the restoration and management of rocky desertification areas.

## 1 Introduction

Plant functional traits are key indicators of species distribution (Engelbrecht et al., 2007; Bohner and Diez, 2020). Their variation reflects adaptation strategies to environmental conditions (Westoby et al., 2002; Reich, 2014; Maynard et al., 2022; Laughlin, 2023) and offers potential for predicting ecosystem responses to global climate change (Díaz et al., 2007; Suding and Goldstein, 2008; Joswig et al., 2022). Derived from these traits, plant strategy theory reveals a fundamental trade-off between rapid resource acquisition and efficient conservation (Reich, 2014; Díaz et al., 2016; Bergmann et al., 2020). This trade-off is captured by the leaf economics spectrum (Wright et al., 2004), a framework extended to stems and roots, forming parallel wood and root economics spectra (Chave et al., 2009; Kong et al., 2019). However, methodological challenges in root trait measurement have constrained research on the root economics spectrum (Freschet et al., 2010), fueling ongoing debate about trait coordination across plant organs at the whole-plant level (Liu et al., 2023; Bai et al., 2025; Da et al., 2025).

Plants rely on the integrated functioning of fundamental organs (leaf, stem, and root) to regulate survival, growth, and reproduction, thereby influencing thier overall performance (Ackerly et al., 2000). The leaf is the primary site of photosynthesis (Bhatla and Lal, 2023). The stem provides structural support, facilitates the transport of water and nutrients, and acts as a storage organ (Taiz et al., 2015). The root system anchors the plant, absorbs water and inorganic ions, and also contributes to storage (Hodge et al., 2009). The synergy among these organs shapes plant growth patterns and ecological strategies (Lambers et al., 2008; Fortunel et al., 2012; Reich, 2014; de la Riva et al., 2016; Liu et al., 2023). However, whether functional traits of leaves, stems, and roots are coordinate across or within species remains debated (de la Riva et al., 2016; Wang et al., 2017; Vleminckx et al., 2021; Fridley et al., 2022). Furthermore, while interspecific comparisons are common, a limited focus on intraspecific trait variation hinders a comprehensive understanding of plant organ functional integration (Chacón-Labella et al., 2023; Bai et al., 2025; Palacio et al., 2025).

The current understanding of the relationship between leaf and stem traits is framed by two competing hypotheses The first, grounded in plant life history theory, proposes a coordinate evolution of leaf and stem traits (Grime et al., 1997). This coordination is attributed to shared construction costs, involving concurrent investments in carbon, nitrogen, and dry matter content in both organs (Freschet et al., 2010). Furthermore, under resource limitation, plants are thought to optimize traits suites to improve resource acquisition, retention, and use efficiency, thereby enhancing stress tolerance and sustaining growth (Méndez-Alonzo et al., 2012; de la Riva et al., 2016; Wang et al., 2017; Kawai and Okada, 2019). In contrast, the leaf-stem trait decoupling hypothesis suggests that these traits evolved under distinct selective pressures. In resource-poor environments, leaves primarily adapt to maximize photosynthetic efficiency (Wright et al., 2007; Bai et al., 2025), while stems evolve to optimize hydraulic conductivity and mechanical support (Baraloto et al., 2010; Fortunel et al., 2012). In more favorable, resource-rich conditions, the imperative for tight trait coordination diminishes, allowing for decoupling to occur (Freschet et al., 2010; Weemstra et al., 2016).

Similarly, the coordination or decoupling of stem and root traits depends on the continuous vascular system (xylem and phloem) (Pratt et al., 2007; Wang et al., 2017) and its adaptation to external environmental pressures (de la Riva et al., 2016; Wang et al., 2017). Under drought conditions, plants modulate water conductivity and carbon allocation to improve water uptake, transport, and conduction efficiency in stems and roots (de la Riva et al., 2016). In extreme drought or nutrient−poor environments, increased resource allocation to stems (for water retention or defense) or to roots (for water and nutrient acquisition), can lead to trait decoupling (Baraloto et al., 2010; Fortunel et al., 2012). Recent studies, however, show that stem and root traits within the same species may be decoupled across different habitats (rocky desertification and non-rocky desertification) with nutrient-use strategies and water-use strategies operating independently (Bai et al., 2025). Thus, further investigation across ecosystems and species is needed to determine whether such decoupling or coordination is driven by environmental stressors.

The relationship between leaf and root functional traits remains a subject of debate, with hypotheses spanning from tight coupling to complete independence. Conclusions often differ depending on the scale of analysis (studied in community and species level). One widely cited hypothesis posits a close functional coordination between leaves and roots, driven by the interdependence of their roles: leaves rely on water and nutrients absorbed by roots, while roots depend on carbohydrates synthesized in leaves (Chapin, 1980; Kerkhoff et al., 2006; Freschet et al., 2010; Reich, 2014; de la Riva et al., 2016). Evidence supporting this view has been reported at the community level (Pérez‐Ramos et al., 2012), whereas species-level analyses have sometimes reached contrasting conclusions (Bai et al., 2025). An alternative perspective suggests that leaf and root traits may be decoupled, particularly under environmental stress. For instance, nutrient limitation or water scarcity can stimulate root growth at the expense of leaf development (Craine and Lee, 2003; Craine et al., 2005; Cordlandwehr et al., 2013; Fort et al., 2013; Bai et al., 2025), or trigger adjustments in root traits without parallel changes in leaf traits (Freschet et al., 2013). A third hypothesis emphasizes trait independence driven by competitive pressures: aboveground light competition favors optimized leaf canopy traits, while belowground competition leads to diversified root foraging strategies (Fortunel et al., 2012). Additionally, hydraulic segmentation may allow stems and roots to endure drought while protecting leaves (Baraloto et al., 2010; Choat et al., 2018). Thus, further research is needed to clarify trait–trait relationships across different organizational levels (species vs. community) and environmental contexts, to elucidate interspecific trait coordination, and to uncover underlying plant adaptation strategies (Zhou et al., 2022; Bai et al., 2025).

*Pseudotsuga sinensis* is a Tertiary relict plant in the genus *Pseudotsuga* (Pinaceae) and an endangered species endemic China (Xiong et al., 2017). Its distributed spans several provinces in China, including Yunnan, Guizhou, Sichuan, Hubei, and Hunan (Xiong et al., 2017; Wen, 2022), often in scattered, fragmented populations (Nong et al., 2024). Pure stands of *Pseudotsuga sinensis* are now rare, through one relatively continuous and intact population persists in a rocky desertification habitat in Weining County, Bijie City, Guizhou Province (covering 373.2 hectares; He et al., 2021). This population provides a valuable opportunity to investigate the coordination of leaf, stem, and root functional traits in plants adapted to karst rocky desertification environments, as well as their integrated strategies for resource acquisition and environmental adaptation. In this study, we used *Pseudotsuga sinensis* from this rocky desertification habitat to examine interspecific relationships among leaf, stem, and root traits. We measured 10 leaf traits related to resource acquisition (light, water, and nutrients) and resistance to environmental stress (**Table 1**, Wright et al., 2004; Reich, 2014; Pérez‐Harguindeguy et al., 2016), along with 20 stem and fine-root traits associated with wood structure, nutrient transport, and defense (**Table 1**, Pratt et al., 2007; Chave et al., 2009; Fortunel et al., 2012; Fridley et al., 2022). Karst rocky desertification environments are characterized by aridity, high-temperatures, and nutrient- deficiency (Wang, 2003; Liu et al., 2011; Xiong et al., 2012). In rocky desertification environments, plants face the need to allocate resources among different functions as various organs respond to distinct stress factors. Trait decoupling allows plants to flexibly adjust the traits of one organ to adapt to specific environmental conditions without significantly altering the functions of another organ (Baraloto et al., 2010; Weemstra et al., 2016; Laughlin, 2023; Bai et al., 2025). Therefore, we hypothesized that the traits of *Pseudotsuga sinensis* leaves, stems, and roots are functionally decoupled.

**Table 1:** The 30 measured traits of plant organ (group).

Trait	Abbreviation	Unit	Group
Leaf area	LA	cm^2^	Leaf
Leaf thickness	LT	mm	Leaf
Specific leaf area	SLA	cm^2^ g^−1^	Leaf
Leaf dry matter content	LDMC	g g^−1^	Leaf
Leaf carbon concentration	LC	mg g^−1^	Leaf
Leaf nitrogen concentration	LN	mg g^−1^	Leaf
Leaf phosphorus concentration	LP	mg g^−1^	Leaf
Leaf potassium concentration	LK	mg g^−1^	Leaf
Leaf calcium concentration	LCa	mg g^−1^	Leaf
Leaf magnesium concentration	LMg	mg g^−1^	Leaf
Wood density	WD	g cm^–3^	Stem
Vessel diameter	*D*_V_	um	Stem
Vessel density	VD	no. mm^–2^	Stem
Theoretical hydraulic conductivity	*K*_t_	kg m^–1^ s^–1^ MPa^–1^	Stem
Stem carbon concentration	SC	mg g^–1^	Stem
Stem nitrogen concentration	SN	mg g^–1^	Stem
Stem phosphorus concentration	SP	mg g^–1^	Stem
Stem potassium concentration	SK	mg g^−1^	Stem
Stem calcium concentration	SCa	mg g^−1^	Stem
Stem magnesium concentration	SMg	mg g^−1^	Stem
Specific root length	SRL	cm g^–1^	Root
Specific root area	SRA	cm^2^ g^–1^	Root
Root dry matter content	RDMC	g g^–1^	Root
Root tissue density	RTD	g cm^–3^	Root
Root carbon concentration	RC	mg g^–1^	Root
Root nitrogen concentration	RN	mg g^–1^	Root
Root phosphorus concentration	RP	mg g^–1^	Root
Stem potassium concentration	RK	mg g^–1^	Root
Stem calcium concentration	RCa	mg g^–1^	Root
Stem magnesium concentration	RMg	mg g^–1^	Root

## 2 Materials and methods

### 2.1 Study site

This study was conducted in the *Pseudotsuga sinensis* Nature Reserve, located in Weining County, Guizhou Province, in southwestern China. The region has a subtropical monsoon climate, with an average annual temperature of 10.5 °C and a mean annual precipitation of approximately 1000 mm. Approximately 88% of the annual rainfall occurs between May and October (Mu et al., 2024). The elevation of the study area ranges from 1800 to 2450 m above sea level. The dominant soil types are yellow-brown soil, calcareous soil, and latosol, with an average soil pH of about 5.50 (Li et al., 2025).

### 2.2 Plot establishment

*Pseudotsuga sinensis* has been reported to be widely distributed in the rocky desertification habitats of Guizhou Province, southwestern China (Li and Xie, 2015; Cui et al., 2025). Rocky desertification is characterized by vegetation coverage of less than 70%, a rock exposure rate exceeding 30%, and an average soil depth below 50 cm (Li et al., 2007). Based on these classification criteria, we established four 20 m × 20 m quadrats in the *Pseudotsuga sinensis* Nature Reserve. Adjacent quadrats were spaced approximately 1 km apart to reduce microclimatic variations. Each quadrat was further subdivided into four 10 m × 10 m subplots to facilitate standardized and scientific sampling.

### 2.3 Sampling

In August 2022, we sampled a total of 24 *Pseudotsuga sinensis* trees (approximately 30 cm in diameter at breast height, DBH) across four 20 m × 20 m quadrats, with 6 trees selected from the center of each quadrat and its nested 10 m × 10 m subplots. Prior to collection, plant sampling was approved by the Weining County Forestry Bureau, Bijie City, Guizhou Province. A total of six trees were sampled per 20 m × 20 m quadrat. Across all four quadrats, a total of 24 trees were sampled. For each tree, samples of leaves, stems, and fine roots were collected. Sun-exposed stems were obtained from the four cardinal directions (east, west, south, and north) using pole pruners. From these stems, healthy, intact, and mature leaves were selected and pooled into a composite sample per tree, stored in a resealable plastic bag. Similarly, stem segments (three stem segments with a diameter ~1 cm) from all direction were combined into another composite sample. Fine roots were sampled by excavating soil approximately 50 cm from the trunk in each cardinal direction. Following the main root, we carefully extracted intact fine roots systems with diameters less than 2 mm, collecting three such systems per direction. Therefore, 4 leaf samples, 12 stem samples, and 12 fine root samples were collected from each individual *Pseudotsuga sinensis* tree. To account for directional variations, samples from the four cardinal directions of each tree were combined into a single composite sample. Consequently, a total of 24 leaf, stem, and root samples were obtained, respectively. All plant samples were placed in a sampling box with ice packs and transported to the laboratory for subsequent analysis.

### 2.4 Trait measurements

For leaf trait measurements, twenty leaves were randomly selected from each *Pseudotsuga sinensis* individual. After removing petioles, the fresh weight of the leaves was determined using a precision electronic balance (accuracy: 0.0001 g). Given the flattened morphology of *Pseudotsuga sinensis* leaves, leaf area (LA, cm^2^) was estimated by calculating the product of leaf length and width at the mid-point (measured using vernier calipers), along with leaf thickness (LT, mm). All leaves were then oven-dried at 70°C for 48 h to obtain their dry weight. The remaining leaf samples were processed by removing petioles, followed by oven-drying under the same conditions. Leaf dry matter content (LDMC, g g^−1^) was calculated as the dry weight to fresh weight ratio. Specific leaf area (SLA, cm^2^ g^−1^) was calculated as the leaf area to dry weight ratio. The dried material was ground into a fine powder (passed through a 2-mm sieve) for subsequent leaf element concentration analysis.

For each tree, four stems were selected. After removing the bark and pith, their wood volume was measured using the water displacement method. The samples were then oven-dried at 70 °C for 72 h, and their dry mass was determined using an electronic balance. Wood density (WD, g cm^−3^) was calculated as the ratio of dry mass to volume. The remaining stem materials were subsequently oven-dried under identical conditions and ground into a fine powder (passed through a 2-mm sieve) for elemental analysis. Next, one side of each stem was smoothed to create a flat transverse surface. Cross-sections were photographed using an Ultra-depth digital microscope (Yiweishike Technology Co., Ltd., Chengdu, China). Five clear images per stem, each showing distinct vessels, were captured in TIFF format at a resolution of 600 dpi, resulting in a total of twenty images for the four stems. ImageJ software (https://imagej.en.softonic.com/ accessed on 22 May 2023) was used to measure, for each image, the number of vessels, the major and minor axis radii, and the field of view area. Vessel diameter (*D*_V_, μm) was calculated using the following formula (Lewis, 1992):


$$D_{V}={\left[32{\left(ab\right)}^{3}/\left(a^{2}+b^{2}\right)\right]}^{1/4}$$

in this formula, a and b represent the semi-major axis and semi-minor axis of the vessel, respectively. The theoretical hydraulic conductivity (*K_t_*, kg m^−1^ s^−1^ MPa^−1^) of the sapwood was determined using the theoretical conductivity approach (Tyree and Ewers, 1991):


$$K_{t}=\left(\pi \rho \right)/128\eta A\left[\sum_{i}^{n}\left(D_{Vi}^{4}\right)\right]$$

in this formula, *ρ* represents the density of water at 25 °C (997.05 kg m^−3^), and *η* represents its dynamic viscosity (0.89 × 10^−9^ MPa s^−1^). *A* and *n* indicate the microscopic field area under observation and the total number of vessels within it, respectively. *D_i_* represents the diameter of an individual vessel.

For the analysis of fine root traits, morphological data from 12 fine roots (selected from four directions) were averaged to represent each sampled tree. The roots were first rinsed with tap water to remove adhering soil, followed by three washes with distilled water. After blotting excess moisture, fresh weight was measured using an electronic balance (accuracy: 0.0001 g). The roots were then submerged in distilled water and carefully spread flat with a soft brush to avoid overlap before being scanned at a resolution of 1200 dpi using a Microtek ScanMaker i850 scanner (Microtek ScanMaker i850, Delhi, India). Subsequently, the samples were oven-dried at 70 °C for 72 h and reweighed. Root length (RL, cm), root diameter (RD, mm), root volume (RV, cm³), and root surface area (RSA, cm²) were analyzed using DJ-GX02 root image analysis software (Dijiang Technology Co., Ltd., Shanghai, China). Derived traits were also calculated: specific root length (SRL, cm g^−1^; RL divided by dry weight), specific root area (SRA, cm² g^−1^; RSA divided by dry weight), root tissue density (RTD, g cm^−^³; dry weight divided by RV), and root dry matter content (RDMC, g g^−1^; dry weight divided by fresh weight). Finally, the dried fine roots were ground into powder (passed through a 2-mm sieve) in preparation for subsequent elemental analysis.

Elemental analysis was performed as follows: carbon (C) and nitrogen (N) concentrations were determined via Dumas-Type combustion using a Vario MAX CN analyzer (Elementar, Gurgaon, India). Phosphorus (P), potassium (K), calcium (Ca), and magnesium (Mg) concentrations were analyzed by inductively coupled plasma atomic emission spectroscopy (ICP-AES; iCAP 7400, Thermo Fisher, Waltham, MA, USA). The N:P ratio, a key indicator of nutrient limitation, was also calculated (Wang et al., 2019).

### 2.5 Data analyses

Data analysis was conducted using the mean values for each selected tree. Prior to analysis, the data were log10-transformed to improve normality, and tests were performed for normality of distribution and homogeneity of variance. To evaluate the associations between different organ traits, we employed Pearson correlation analysis. To investigate whether consistent economic spectra exist among *Pseudotsuga sinensis* organs in karst rocky desertification forests and to examine inter-organ coordination patterns, we performed principal component analysis (PCA) to assess correlations among different organ traits. This approach allowed us to determine whether: (1) most functional variation is concentrated along a single dimension, (2) different organs show similar loading patterns, or (3) specific organ combinations explain independent and significant portions of the total functional inertia (Vleminckx et al., 2021). The PCA axis loadings were subsequently used as indicators of organ economic strategies. Furthermore, through correlation analysis, we conducted a paired analysis of the distribution of species scores along the first principal component axis in different organs. we quantified the degree of association between trait spectra of different plant organs (Freschet et al., 2010). All statistical analyses and graphical visualizations were performed in R software (version 4.4.0).

## 3 Results

**Figure 1:**
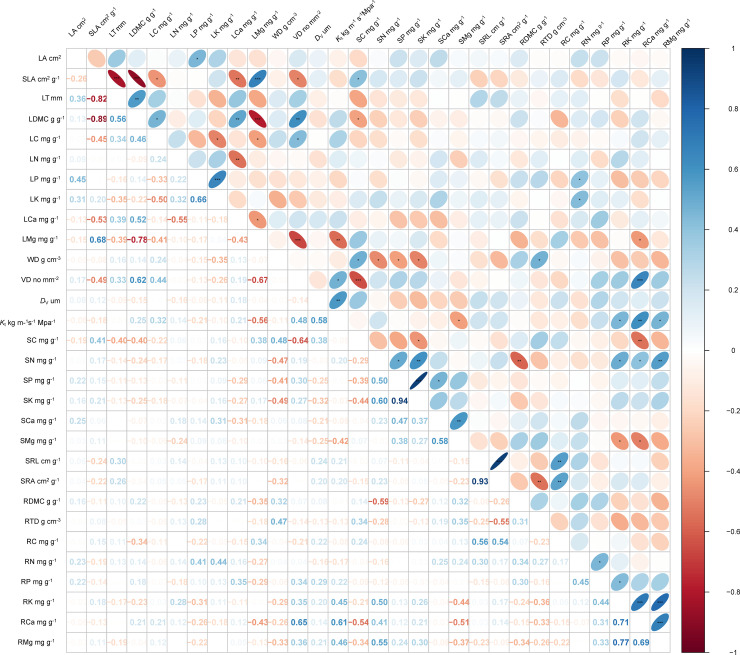
Pearson’s correlation matrix between *Pseudotsuga sinensis* leaf, stem, and root traits. Abbreviations for traits are given in **Table 1**. *p < 0.05; **p < 0.01, ***p < 0.001.

Analysis of plant trait covariation patterns revealed distinct correlation characteristics at the organ level. Intrinsic functional traits within organs (leaves, stems, or roots) consistently demonstrated strong interdependence, whereas inter-organ trait relationships exhibited significantly weaker correlations (**Figure 1**).

Specifically, we observed: (1) significant positive correlations between LDMC (*r* = 0.62, *p* < 0.01) and LC (*r* = 0.44, *p* < 0.05) with VD; (2) a significant negative correlation between VD and SLA (*r* = −0.49, *p* < 0.05); (3) a positive correlation between SLA and SC (*r* = 0.41, *p* < 0.05), but a negative correlation between LDMC and SC (*r* = −0.40, *p* < 0.05); (4) negative correlations of LMg with VD (*r* = −0.67, *p* < 0.001), *K*_t_ (*r* = −0.56, *p* < 0.01), and RCa (*r* = −0.43, *p* < 0.05); (5) a negative correlation between *K*_t_ and SMg (*r* = −0.42, *p* < 0.05); (6) positive correlations of LP (*r* = 0.41, *p* < 0.05) and LK (*r* = 0.44, *p* < 0.05) with RN; (7) a positive correlation between WD and RDMC (*r* = 0.32, *p* < 0.05); (8) positive correlations between VD and RCa (*r* = 0.65, *p* < 0.001), and between *K*_t_ and RK (*r* = 0.45, *p* < 0.05), RCa (*r* = 0.61, *p* < 0.01), and RMg (*r* = 0.46, *p* < 0.05); and (9) positive correlations of SN with RK (*r* = 0.50, *p* < 0.05), RCa (*r* = 0.41, *p* < 0.05), and RMg (*r* = 0.55, *p* < 0.01).

**Figure 2:**
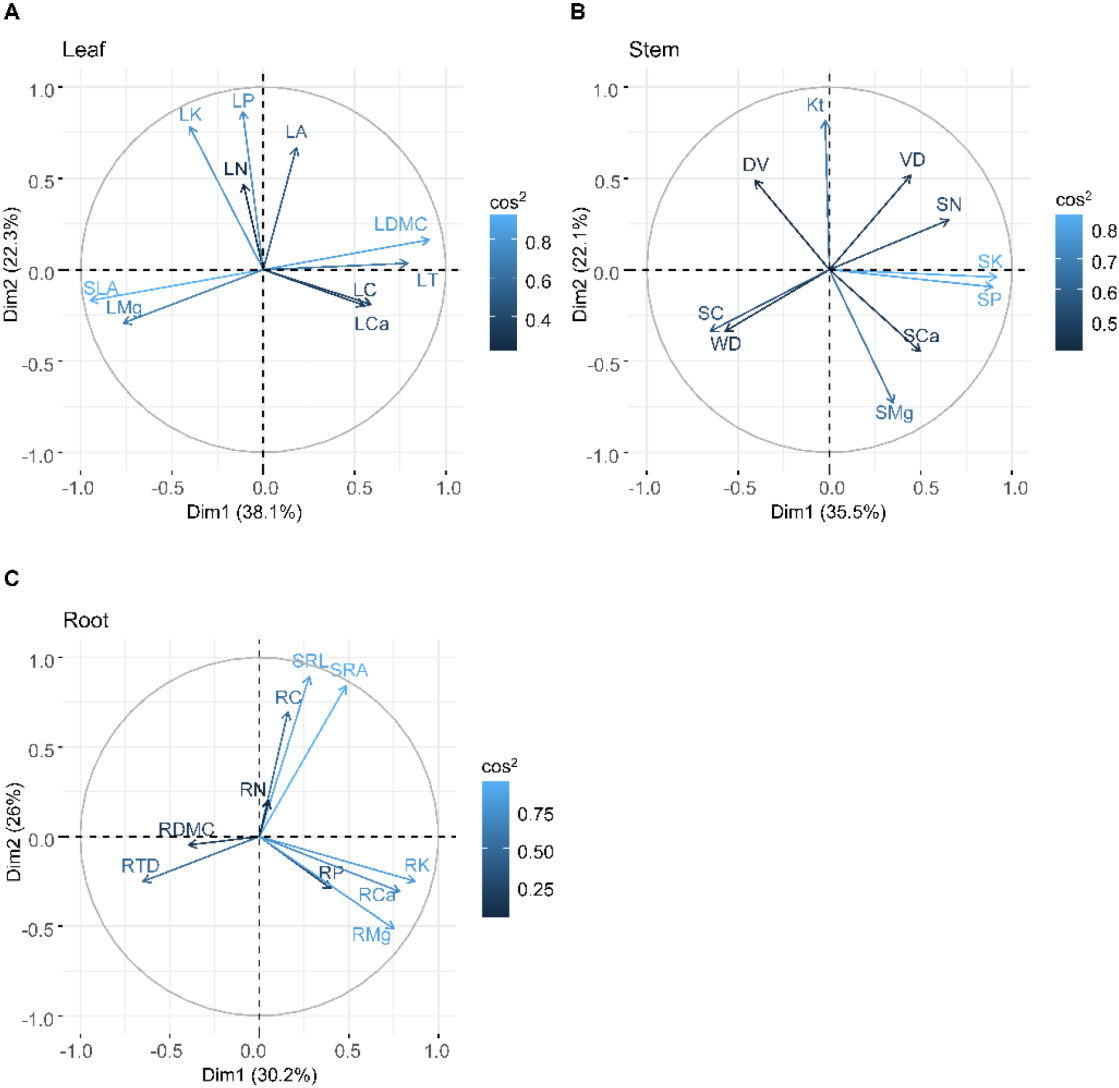
Principal components analysis of leaf **(A)** stem **(B)** and root **(C)** traits. Abbreviations for traits are given in **Table 1**.

Principal component analysis (PCA) of leaf, stem, and root traits in *Pseudotsuga sinensis* from a rocky desertification forest revealed distinct patterns of trait variance (**Figure 2**). The first two principal axes accounted for varying proportions of total variance across tissues (leaves: PCA 1, 38.1%, PCA 2, 22.3%; stems: PCA 1, 35.5%, PCA 2, 22.1%; roots: PCA 1, 30.2%, PCA 2, 26.0%). For leaves, traits associated with water conservation and carbon storage (LDMC, LT, LC, LCa) loaded positively on PCA 1, while light acquisition traits (SLA, LMg) showed negative loadings (**Figure 2A**). Nutrient-related traits (LN, LP, LK) were positively associated with PCA 2, indicating that leaf water/light strategies are largely independent of nutrient utilization. Stems analysis demonstrated positive PCA 1 loading for nutrient traits (SN, SP, SK) versus negative loadings for carbon storage/defense traits (SC, WD). Water transport efficiency traits aligned with PCA 2, revealing an orthogonal axis between nutrient allocation and hydraulic efficiency (**Figure 2B**). Root PCA showed that traits related to nutrient demand (RP, RK, RCa, RMg) loaded positively on PCA 1, contrasting with defense traits (RDMC, RTD). Soil nutrient acquisition traits (SRL, SRA, RC) correlated with PCA 2, suggesting independent variation between root nutrient foraging and internal nutrient requirements.

**Figure 3:**
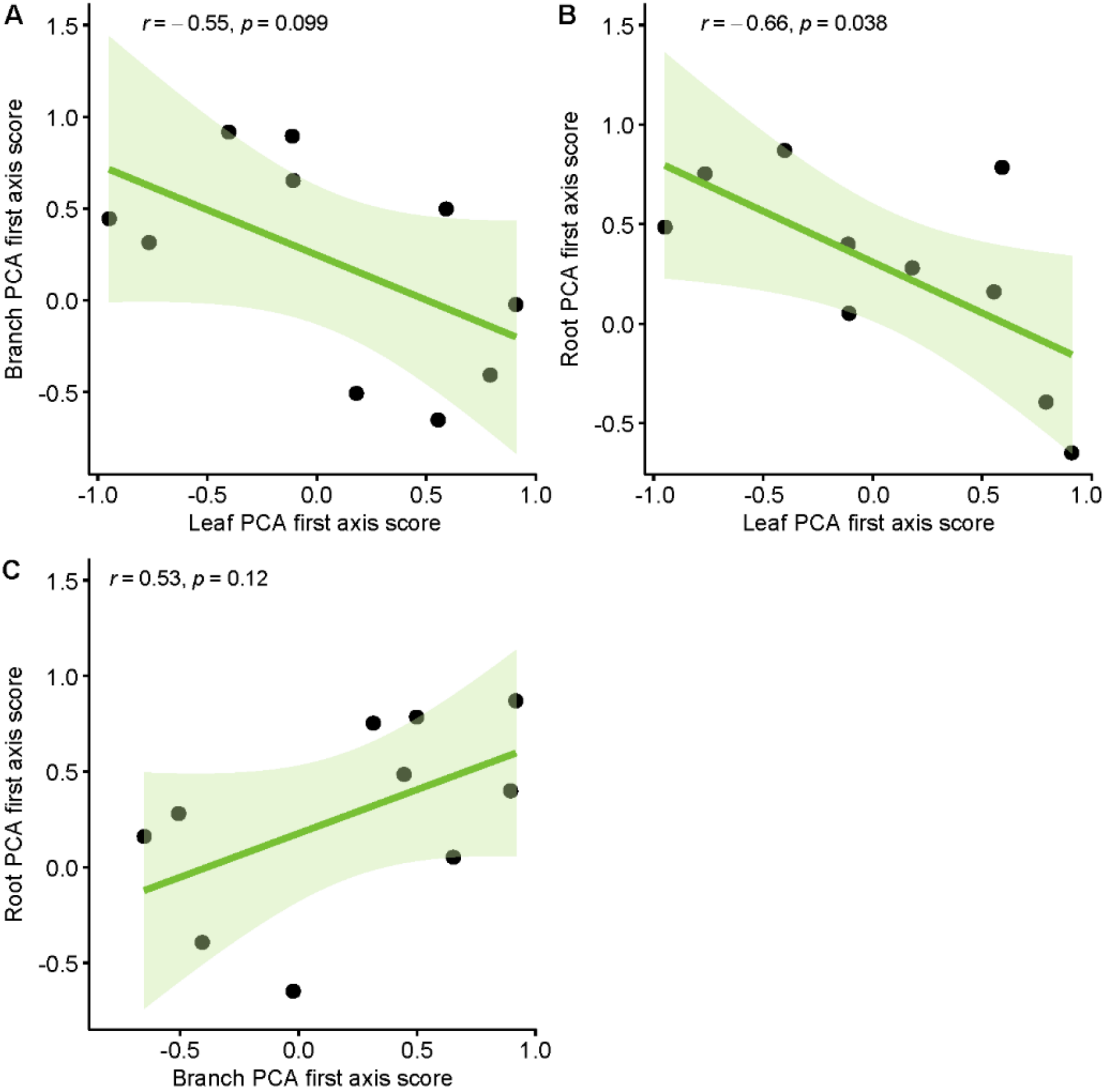
Correlations between economics spectra, as indicated by PCA first axis score, of different plant parts.

These findings partially supported our hypothesis, demonstrating a trade-off relationship between leaf and root economic spectra in rocky desertification forests, while stem traits remain independent of both. Principal component analysis (PCA) integration further confirmed a significant negative correlation between leaf traits and fine root traits along the first principal axis (**Figure 3B**). In contrast, no significant correlations were observed between stem traits and either leaf or fine root traits (**Figures 3A, C**).

## 4 Discussion

Plant strategies give rise to a suite of interrelated traits shaped by functional trade-offs in resource allocation. However, understanding these trade-offs from a whole-plant perspective has been hindered by limited and inconsistent knowledge of belowground root traits and their coordination with other organs (Westoby et al., 2002; Baraloto et al., 2010; Reich, 2014; Silva et al., 2018; Luo et al., 2024). Consequently, elucidating inter-organ trade-offs remains both a central focus and a persistent challenge in plant ecology (de la Riva et al., 2016; Wang et al., 2017; Liu et al., 2023; Laughlin, 2023; Zhao et al., 2024; Bai et al., 2025). In this study, we analyzed 30 functional traits across leaves, stems, and roots of *Pseudotsuga sinensis*. Contrary to our hypothesis of widespread organ decoupling, the results revealed a tight coordination between leaf and root traits, whereas leaf-stem and stem-root associations were decoupled. This pattern indicates a potential resource allocation trade-off between leaves and roots in *Pseudotsuga sinensis*.

Contrary to the assumptions of life history theory (Grime et al., 1997), our study found no significant correlation between the leaf and stem economic traits of *Pseudotsuga sinensis* in the karst rocky desertification region. This finding indicates that, within the study area, the leaf economics spectrum (Wright et al., 2004) and the stem economics spectrum (Chave et al., 2009) operate independently of each other. Such decoupling of leaf and stem economic spectra aligns with findings from studies on tropical, subtropical, and karst rock desertification tree species (Baraloto et al., 2010; Wang et al., 2017; Vleminckx et al., 2021; Bai et al., 2025). This demonstrates how plants adapt to the nutrient deficit and arid conditions of karst rocky desertification environments through differentiated resource allocation strategies in leaves and stems. In the high-temperature, arid, and nutrient-poor environment of rocky desertification, plants likely adapt by thickening their leaves (to reduce water loss) and enhancing nutrient retention in the foliage. However, such adaptations may not necessarily require simultaneous changes in stem investment (Bai et al., 2025). In contrast, studies conducted in subtropical Pacific islands ecosystems (Ishida et al., 2008), Mediterranean forests (de la Riva et al., 2016), and Neotropical forests (Fortunel et al., 2012) have identified coordinated relationships in leaf-stem trait spectra. This contrast suggests that trait coordination among leaves, stems, and roots is not a universal pattern, with variations depending on the specific constraints of each ecosystem (Kembel and Cahill, 2011; Wang et al., 2017). Successful strategies within a given environment are shaped by the abiotic and biotic filters that act upon the local plant community (Cornwell and Ackerly, 2009; Kraft and Ackerly, 2010).

For stem and root traits in *Pseudotsuga sinensis*, we found no significant correlation. This indicates that the leaf economic spectrum (Wright et al., 2004) and the root economic spectrum (Roumet et al., 2016) operate independently. Our results do not support the hypothesis of continuously coordinated resource allocation between stems and roots via vascular tissues (Tyree and Ewers, 1991; Pratt et al., 2007). These findings are consistent with observations in *Quercus rehderiana* from karst rocky desertification regions (Bai et al., 2025). This may be attributed to the fact that plants in arid and barren soils tend to develop denser root systems to enhance water and nutrient uptake, while stem traits may prioritize a balance between hydraulic safety and efficiency (Reich, 2014; Weemstra et al., 2016; Kong et al., 2019; Bai et al., 2025). In contrast, studies on shrubs in Shaanxi Province, China, have reported shared chemical properties and structural traits between stems and roots, along with interconnected resource allocation (Kerkhoff et al., 2006; Freschet et al., 2010; Fortunel et al., 2012; Wang et al., 2017). Thus, no universal consensus exists on stem-root relationships across different ecosystems.

Our study found a significant negative correlation between leaf and root traits. This contrasts with earlier reports of positive correlations in shrubs from Shaanxi Province, China (Wang et al., 2017) and herbaceous plants in Mediterranean grasslands of southern France (Pérez‐Ramos et al., 2012). Although leaves and roots are functionally interdependent during growth and development (Chapin, 1980). Leaf function relies on water and nutrients absorbed by roots, while root growth depends on carbohydrates produced by leaves (Chapin, 1980; Kerkhoff et al., 2006; Freschet et al., 2010; Reich, 2014; de la Riva et al., 2016). Principal component analysis in this study revealed a trade-off in structural resources allocation for defense strategies between the two organs. This trade-off is reflected in the balance of resource allocation between photosynthetic carbon acquisition and water-nutrient absorption. In karst rocky desertification habitats, drought and nutrient deficiency directly drive the allocation of photosynthetic products towards root investment to enhance water and nutrient uptake, while leaves adopt a more conservative strategy (characterized by low SLA) to improve resource retention (Freschet et al., 2010; Reich, 2014; Wang et al., 2026). The trade-offs between leaf and root traits in karst rocky desertification areas reveal plant resource allocation strategies and stress resistance adaptation mechanisms. This provides critical scientific insights for understanding the eco-physiological basis of vegetation restoration in fragile karst ecosystems, selecting suitable species, and optimizing ecological restoration strategies. Nevertheless, the relationship remains inconsistent across studies, with several reporting no correlation between leaf and root traits (Liu et al., 2010; Fortunel et al., 2012; Bai et al., 2025).

Currently, the relationship between leaves and stems has received extensive attention and has been most thoroughly studied (Baraloto et al., 2010; Méndez-Alonzo et al., 2012; de la Riva et al., 2016; Kawai and Okada, 2019). While some studies have integrated roots into plant-level trade-off analyses, most focus on variations across ecosystems, communities, and species, overlooking intraspecific variation in whole-plant strategy trade-offs (Garnier et al., 2001; Kraft et al., 2015; Da et al., 2025). Therefore, future research on leaf-stem-root relationships should encompass a broader range of ecosystems and conduct deeper analyses across multiple levels (community, interspecific, and intraspecific scales) to achieve a more comprehensive understanding of trait associations among plant organs.

## 5 Conclusions

In this study, we identified a trade-off in the allocation of defense structural compounds between leaves and roots in *Pseudotsuga sinensis*, as indicated by a significant negative correlation between their respective traits. The relationships between leaf and stem and between stem and root were decoupled due to divergent resource strategies related to water, nutrients, and light. However, research on intraspecific trait associations among leaves, stems, and roots remains limited, constraining our understanding of their functional roles across different ecosystems or species. Therefore, future studies across diverse ecosystems (particularly at the intraspecific level) are needed to investigate trait coordination among plant organs and to draw reliable conclusions regarding functional integration. The results indicate that different plant organs adopt distinct resource strategies (e.g., water, light, nutrients) to cope with environmental conditions. This study provides theoretical insights for the cultivation and management of trees in rocky desertification regions.

## Data Availability

The datasets presented in this study can be found in online repositories. The names of the repository/repositories and accession number(s) can be found below: DOI: 10.5061/dryad.zkh1893r4.
